# Antegrade intramedullary Kirschner-wire fixation of displaced metacarpal shaft fractures

**DOI:** 10.1007/s00068-017-0836-0

**Published:** 2017-09-14

**Authors:** E. M. van Bussel, R. M. Houwert, T. J. M. Kootstra, M. van Heijl, D. Van der Velde, Ph. Wittich, J. Keizer

**Affiliations:** Department of Traumatology, St Antonius Hospital, Koekoekslaan 1, 3435 CM Nieuwegein, The Netherlands

**Keywords:** Metacarpal, Shaft, Fractures, Internal fixation, Intramedullary, Kirschner wire, Minimal invasive, Patient-related outcome

## Abstract

**Purpose:**

The objective of this study was to analyze complications and patient-related functional outcome after antegrade intramedullary Kirschner-wire fixation of metacarpal shaft fractures.

**Methods:**

All consecutive patients treated from January 2010 until December 2015 were retrospectively analyzed using patient logs and radiographic images. Indications for operative fixation were angulation > 40°, shortening > 2 mm, or rotational deficit. Complications were registered from the patient logs. Functional outcome was assessed with the Patient-rated wrist/hand evaluation (PRWHE) and Disabilities of the Arm, Shoulder, and Hand score (DASH) questionnaire both ranging from 1 to 100 after a minimum follow-up of 6 months.

**Results:**

During the study period, 34 fractures of 27 patients could be included. Mean outpatient follow-up was 11 weeks (range 4–24 weeks). The mean interval for functional assessment was 30 months (range 8–62 months) and 19 patients (70%) responded to the questionnaires. During outpatient follow-up, all fractures proceeded to union with no signs of secondary fracture dislocation or implant migration. One re-fracture after a new adequate trauma was seen and one patient underwent tenolysis due to persistent pain and impaired function. In 26 cases (81%), the K-wires were removed of which 23 (68%) were planned removals. Functional outcome was excellent with mean PRWHE and DASH scores of 7 and 5 points, respectively.

**Conclusions:**

If surgical treatment for metacarpal shaft fractures is considered, we recommend antegrade intramedullary K-wire fixation. This technique results in low complication rates and excellent functional outcome.

## Introduction

Fractures of the metacarpal bones account for a significant part of fractures in the hand; percentages up to 40% are described in the literature with a shaft neck ratio of roughly 1:2 [1]. These fractures are frequently observed in young and active adults. Fractures of the metacarpal shaft are commonly observed after a punch or direct trauma. Most of these fractures can be treated conservatively with an intrinsic plus position cast. Surgical treatment is indicated for unstable fractures, large dislocations or shortenings, as well as malrotations and communitive fractures [2].

When surgical intervention is considered, multiple techniques are advocated in the literature such as transverse pinning, minifragment lag screws, and plate fixation [3, 4]. Although multiple studies have shown that plate fixation leads to a very rigid fixation, this technique requires significant soft-tissue dissection. Multiple studies have shown that complications are not uncommon after this technique. Complications such as infections, adhesions, and stiffness might be related to the relatively large incision [5, 6].

An alternative surgical technique is the insertion of one or multiple intramedullary K-wires through a minimal invasive incision in the metacarpal shaft. Both durations of the surgical procedure as the iatrogenic tissue damage might be reduced. This might result in lower complication rates and improved functional outcome while maintaining fracture reduction. This technique was already described in 1976 by Foucher et al. [7], but not many studies focusing solely on metacarpal shaft fractures have been published since then [8–10]. Although fracture healing seems excellent in the available literature, the biggest hiatus might be patient-related functional outcomes, especially after the first few weeks of bone healing. The aim of this study was to analyze complications and patient-related functional outcomes longer than 6 months post-operatively after antegrade intramedullary K-wire fixation of metacarpal shaft fractures.

## Materials and methods

This study was performed according to the Institutional Review Board (IRB) medical ethics standards. The local medical ethical committee analyzed the study protocol and found IRB approval unnecessary due to the non-invasive character of the questionnaires.

### Study design

A single center retrospective observational cohort was defined. The study was performed in a large level 2 regional teaching hospital (St. Antonius Hospital Nieuwegein/Utrecht). All consecutive patients who underwent fixation of an acute metacarpal shaft fracture (within 3 weeks after trauma) with one or more intramedullary Kirschner wires (K-wires) from January 2010 until December 2015 were retrospectively reviewed and invited to participate in this follow-up study. Later cases were not included to obtain follow-up of at least 6 months. All patients included in this study were identified by chart review using diagnosis treatment codes. Indications for operative fixation were angulation > 40°, shortening > 2 mm, or rotational deficits. The following baseline characteristics were collected: gender, age, trauma mechanism [punch, (down) fall, or other external force], side, dominance of the affected hand, smoking, diabetes, open or closed fracture, degree of radiographical angulation, main indication for surgery, and days until surgery.

### Surgical technique

**Fig. 1:**
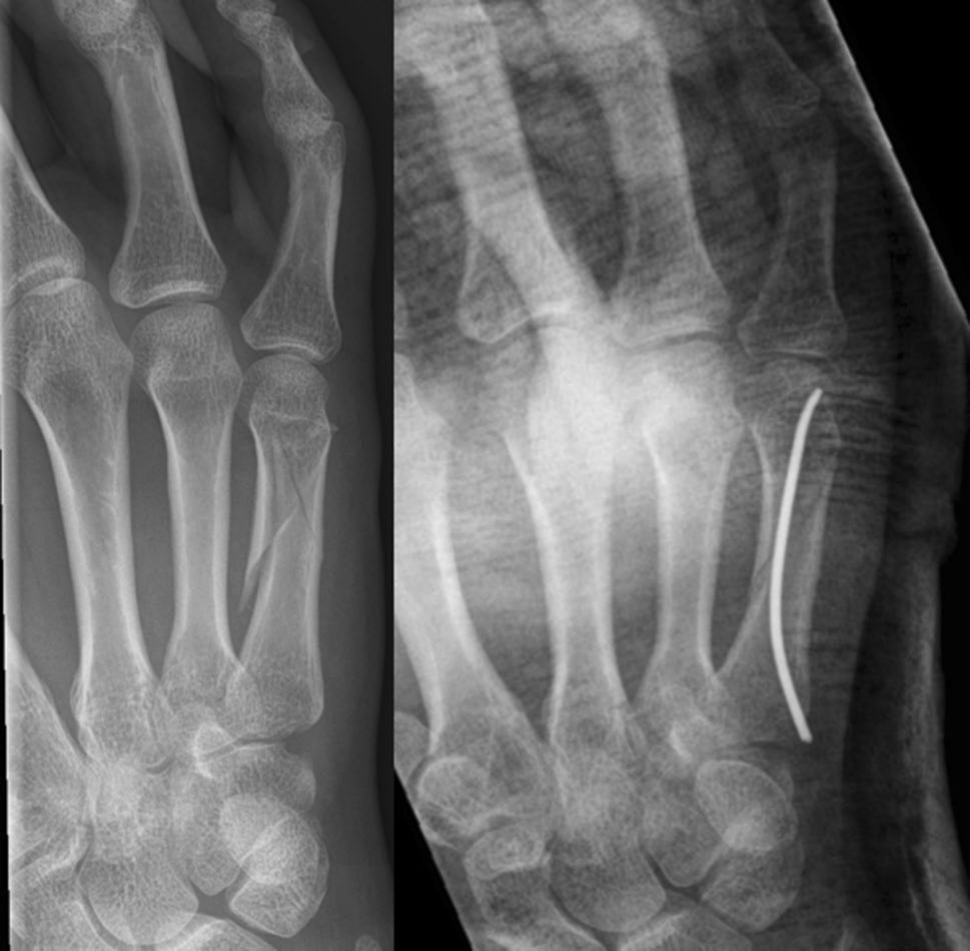
Pre- and postoperative image of short K-wire fixation

**Fig. 2:**
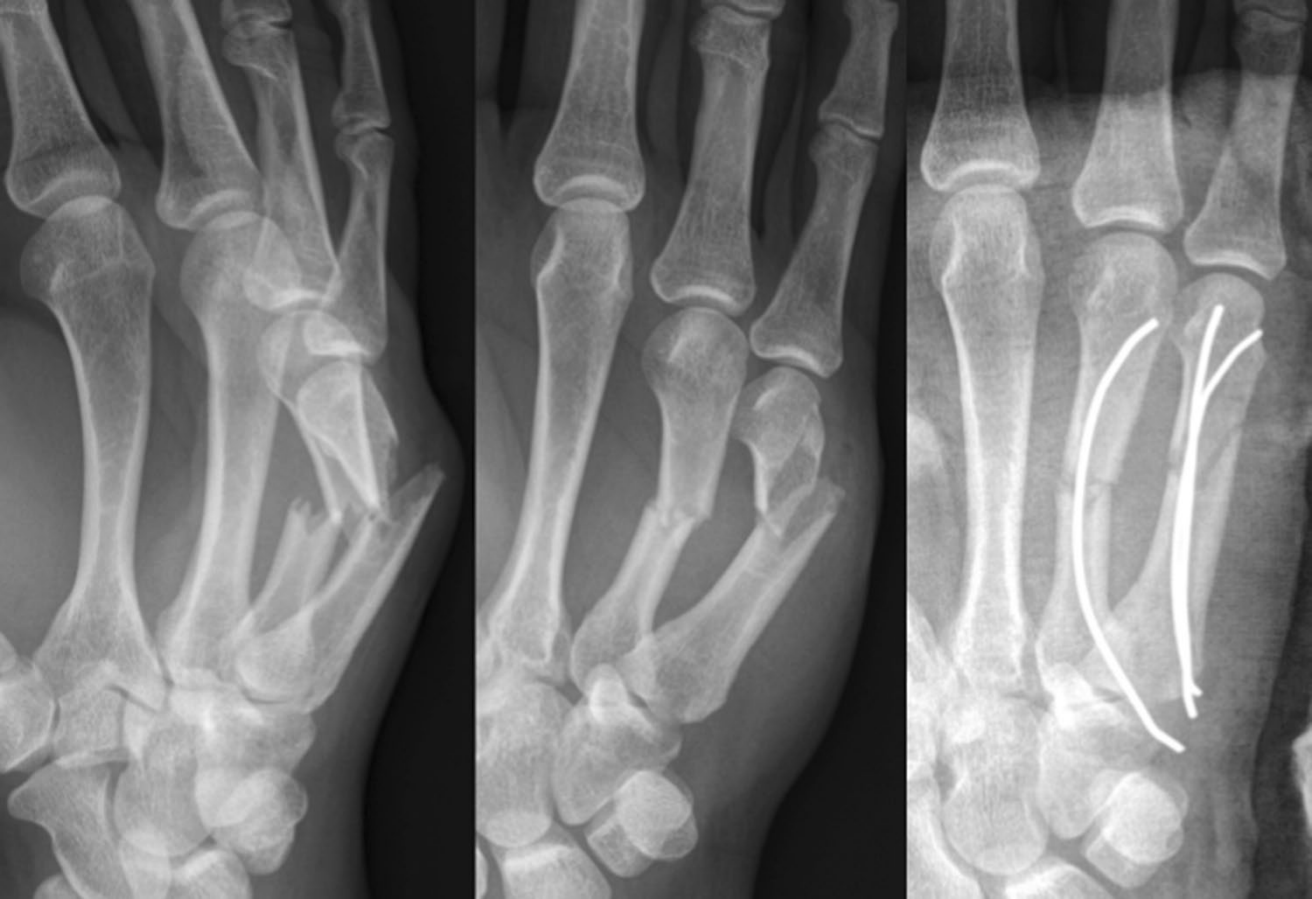
Pre- and postoperative images of long subcutaneous K-wires

Surgical procedures were performed by different trauma surgeons with various levels of experience as well as supervised residents. All patients received pre-operative antibiotic prophylaxis (Cefazolin 2 gram intravenous), while surgery was performed either under loco-regional anesthesia or under general anesthesia. Use of a tourniquet was based on the surgeon’s preference. After sterile exposure, closed reduction was attempted. If closed reduction was unsuccessful, open reduction was performed. Alignment was checked under fluoroscopy. A small skin incision was made at the base of the affected metacarpal bone—at the ulnar side for the fourth and fifth metacarpal bones and ulnar or radial side for the third metacarpal bone—followed by opening of the bony cortex of the metaphysis with a 2.0–2.4 mm drill. A prebended 0.8–1.5 mm K-wire was then intramedullary inserted in the metacarpal bone passing the fracture under fluoroscopy. Final fracture reduction was done by manipulation of the inserted intramedullary K-wire. Stability and position of the K-wire were assessed under fluoroscopy. In case of insufficient stability, a second prebended K-wire was inserted through the same cortical opening. If necessary, a second drill hole was created at the contralateral side of the base of the metacarpal bone. Depending on the surgeon’s preference, the K-wires were cut at the level of the periost for permanent intramedullary fixation (Fig. 1) or subcutaneously to facilitate implant removal (Fig. 2). The skin was closed using absorbable sutures. Patients received either a compression bandage for 24 h or a dorsal blocking cast.

### Postoperative treatment

Patients received dorsal cast immobilisation for a period of 3 weeks or a compression bandage for 24 h with direct functional aftercare. Conventional radiographic images were performed post-operatively. Patients were referred to a specialised hand physiotherapist for guided mobilisation when full function was not seen within 2 weeks after surgery or 2 weeks after cast immobilisation. During follow-up, radiographic, function, and wound control were performed in the outpatient department. Patients were released from outpatient department follow-up after full recovery. Therefore, each patient received clinical follow-up.

### Implant removal

All subcutaneously buried K-wires were removed after 6 weeks. If possible this procedure was performed under local anesthesia, the periosteal buried K-wires were only removed on indication.

### Outcome assessment

All clinical logs were analyzed for short-term postoperative recovery of function and pain during outpatient follow-up. Potential complications were registered like the presence of non- or malunion, secondary fracture dislocation, implant migration, impaired function at the end of clinical follow-up, dysesthesia, infections, and unplanned implant removal. Malunion was defined as a rotational deficit in combination with complete radiographic bony healing. Non-union was defined as lack of radiologic bony healing 6 months after surgery with clinical evidence of pain and/or motion at the fracture site. “Wound healing problems” were defined as any deviation in the postoperative healing process, but without the need for additional pharmacological or surgical intervention. A superficial wound infection was defined as a clinical suspicion of a wound infection based on redness and pus and/or fever in combination with the necessity of antibiotic treatment. A deep wound infection included the characteristics of a superficial wound infection with additional necessity of incision and irrigation in the operating room.

All patients were contacted and invited to participate in a mid-term functional evaluation using the PRWHE (patient-rated wrist hand evaluation) and DASH (Disabilities of the Arm, Shoulder and Hand) questionnaires. Both questionnaires have a score ranging from 0 to 100. In the PRWHE, pain and disability are separately tested with scores ranging from 0 to 50. In the DASH questionnaire, disability is tested by 30 questions next to an optional high-performance score. For both questionnaires, the official Dutch translations were used [11–13].

### Sub-analysis of the fifth metacarpal shaft fractures

The fifth metacarpal shaft fractures were compared to the other metacarpal fractures in separate sub-analyses. All baseline and perioperative parameters, as well as complications and functional outcome, were analyzed.

### Statistical analysis

Baseline characteristics, differences in surgical treatment such as the number of K-wires and type of anesthesia, and differences in postoperative care were analyzed concerning their potential statistical association with the described complications and patient-related outcomes. In addition, the patient-related outcome scores were also analyzed concerning overall results and their statistical association with the recorded complications. Statistical analysis was done with IBM SPSS statistics (version 22.00-2013). For continuous variables, ranges and if appropriate standard deviations (SD) were reported.

## Results

### Inclusion of study population

During the study period, 34 shaft fractures in 27 patients could be included. No patients were lost to follow up in the outpatient department. The mean regular outpatient follow-up was 11 weeks (range 4–24 weeks). The mean interval for mid-term functional assessment was 30 months (range 8–62 months) after primary surgery with a 70% response rate or both questionnaires.

### Baseline characteristics

The baseline characteristics are shown in Table 1. Most patients were young men with a metacarpal shaft fracture after a punch. Twenty fractures were located in the fifth metacarpal bone, and nine fractures in the fourth metacarpal of which two had an additional fracture in the third metacarpal bone. No shaft fractures were seen located in the second metacarpal bone in this study group. In 23 fractures (72%), the K-wires were inserted with the intention of subsequent planned removal by protruding the wires in the subcutaneous tissue for easy removal. In the other nine fractures, the K-wires were cutoff at the level of the periost for a permanent fixation. The mean time of the total procedure from start of anesthesia until the endo of the surgical procedure was 22 min (SD 9). Open fracture reduction was necessary in one fracture. A total of nine patients received physiotherapy due to suspected impaired function during outpatient follow-up. Functional aftercare was initiated in ten cases. A postoperative plaster immobilisation was significantly more frequent given after a single intramedullary K-wire fixation compared to two intramedullary K-wires (82 vs 40%) (*p* = 0.02).

**Table 1:** Baseline characteristics

Characteristics	Total patients (*n* = 27)
Male	21 (78%)
Age in years	25 (SD 11)
Patient characteristics	
Right hand	21 (78%)
Smoking	6 (22%)
Diabetes	1 (4%)
Trauma mechanism	
Punch	11 (41%)
Downfall	6 (22%)
Other (direct) force	10 (37%)

Characteristics	Total fractures (*n* = 32)
Metacarpal	
Third	3
Fourth	9
Fifth	20
Main surgical indication	
Angulation	26 (81%)
Rotation	3 (9%)
Shortening	3 (9%)
Pre-operative days	8 (SD 4)
Surgery time in minutes	22 (SD 9)
Number of K-wires	
I	22 (69%)
II	10 (31%)
Postoperative cast	22 (69%)
Physiotherapy	12 (38%)

*SD* standard deviation

### Complications and implant removal

During outpatient follow-up, all 32 fractures resulted in radiographic and clinical union with no signs of secondary fracture dislocation or implant migration. No cases of malunion were observed. One patient was subject to a new trauma resulting in a re-fracture of the radiographically healing bone 6 weeks after primary surgery and 2 days after removal of the K-wires.

One patient underwent tenolysis of the extensors due to persistent complaints of pain and functional impairment 4 months after removal of the K-wires. Relief of complaints, but no full recovery of pain was seen after the tenolysis in this patient. One other patient did not reach full function at the end of outpatient department follow-up, with an accepted flexion lag of 10°. Two patients experienced persistent local dysesthesia in the area of proximal incision after 11 weeks of follow-up. Complications were not significantly associated with the baseline characteristics, the number of k-wires, or the type of aftercare.

In 26 fractures (81%), the K-wires were removed after a mean of 6 weeks (Table 2), of which 23 removals were planned. Three of the seven fractures with buried K-wires—intended to be permanent—were eventually removed due to pain or stiffness. In all three patients, the K-wire removal resulted in complete relief of complaint. As depicted in Table 2, the buried K-wires were removed under general anesthesia in all three cases, contrary to the planned K-wire removals which were done under local anesthesia in 68% of the cases.

**Table 2:** Removal of K-wires

Characteristics	Short K-wires (*n* = 7)	Long w-wires (*n* = 25)
Number (% total)	7 (22%)	25 (78%)
Removed (% subgroup)	3 (43%)	25 (100%)
Weeks until removal	20 (SD 10)	6.7 (SD 4)
Anesthesia		
General	3 (100%)	8 (32%)
Local	0 (0%)	17 (68%)
Surgery time in minutes	25 (SD 13)	7 (SD 3)

*SD* standard deviation

### Functional outcome

Table 3 shows the functional outcome using the PRWHE and DASH questionnaires. Mean follow-up of both questionnaires was 30 months (SD 17 months) with a response rate of 70%. The average score for the PRWHE and DASH questionnaires was, respectively, 7 and 5 out of a maximum score of 100, with a higher score for pain than for disability in the PRWHE questionnaire. The five patients with the highest DASH and PRWHE scores included the patient with a re-fracture and the patient who underwent tenolysis.

**Table 3:** PRWHE and DASH questionnaire scores

Patient-rated wrist/hand evaluation (PRWHE)	
Number of responses (*t* = 51)	19 (70%)
Total score (0–100)	7 (range 0–37)
Pain score (0–50)	4 (range 0–24)
Disability score (0–50)	2 (range 0–13)
The Disabilities of the Arm, Shoulder and Hand (DASH) score	
Number of responses (*t* = 51)	19 (70%)
Total score (0–100)	5 (range 0–28)

### Sub-analysis of the fifth metacarpal fractures

No differences were found for the incidence of complications, nor the functional outcome as reported by the DASH and PRWHE questionnaires (Table 4).

**Table 4:** Fifth metacarpal versus other (third and fourth) metacarpal shaft fractures

Characteristics	Fifth	Other	Significance* (*r*)
Number	21	11	
Surgery time in minutes	24 (SD 10)	18 (SD4)	*r* = 0.02
Fractures with two K-wires	9	1	*r* = 0.05
Postoperative cast	12	10	*r* = 0.05
Treatment with a short K-wire	7	0	*r* = 0.03
DASH	4.6 (SD 8)	5.1 (SD 6.1)	*r* = 0.9
PRWHE	6.9 (SD 13.3)	8.3 (SD 10.2)	*r* = 0.8

*SD* standard deviation

*Significance *r* = Independent student *T* test or Pearson Chi–Square test

## Discussion

This study demonstrates uncomplicated bone healing after intramedullary K-wire fixation of displaced metacarpal shaft fractures with low complication rates in a selected group of patients. Patient-related functional outcome scores showed excellent function of the injured hand. Although promising results of surgical treatment could be reported, conservative treatment still remains the gold standard for the great majority of patients with metacarpal shaft fractures in our hospital.

To our knowledge, this is the first study focusing solely on metacarpal shaft fractures that has not only analyzed the short-term complications and outcome, but also the results of two well-known PROMs after a significant postoperative period of 30 month average. In addition, several clinical lessons could be conducted from this retrospective cohort regarding type of anesthesia, aftercare, and implant removal. First, surgical intervention done under loco-regional anesthesia was well tolerated in this cohort as none of these patients were converted to general anesthesia. Second, functional aftercare is a safe treatment option as it did not result in higher short-term complication rates. Third, periostal positioning of K-wires resulted in unplanned implant removal in half of the patients. In contrast, cutting off the K-wires subcutaneously resulted in a low rate of wound complications and easy implant removal (68% under local anesthesia in the outpatient department). Therefore, these results support operative treatment under loco-regional anesthesia with functional aftercare. It is recommended to cut off the implant subcutaneously and routinely remove the implant in the outpatient department.

The incidence of our complications appears to be lower compared to other frequently used techniques such as plate fixation and transverse pinning [3, 5, 8]. Bone-healing disorders after surgical treatment of metacarpal shaft fractures in general are rare. Functional limitations are more frequently encountered, probably due to postoperative adhesions or scar tissue. The recently published systematic review of Greeven et al. provides a clear overview of the literature and complication rates of both transverse pinning and plate fixation of metacarpal shaft fractures [3]. Just like intramedullary fixation, transverse pinning is characterized by minimal soft-tissue injury, but shows a high incidence of (pin tract) infections up to 25%. This is probably due to the extra-cutaneous ends of the pins. Although not mentioned in the review but imaginable based on the transverse technique, transverse fixation limits the possibility of functional aftercare and could theoretically lead to more stiffness and pain of the affected hand. The other method reviewed by Greeven et al. is fixation using a plate. As the review illustrates, plate fixation seems a reliable method of fracture fixation with the opportunity of direct functional aftercare. However, plate fixation seems prone to high rates of stiffness, adhesions, and a higher rate of surgical re-interventions (up to 14%). These soft-tissue complications after plate fixation are observed in other studies as well [5, 6]. Our findings suggest that antegrade intramedullary K-wire fixation with subcutaneous burying of K-wire ends does not bear the negative characteristics of transverse pinning nor open reduction and plate fixation. This finding is supported by other studies focusing on antegrade intramedullary K-wire fixation of shaft fractures and literature of metacarpal fractures in general [3, 8–10, 14–17].

We were able to report the mid-term functional outcome using the validated patient-related outcome measurement tools (PRWHE and DASH) in 70% of the study population after 30 months on average. The results of these PROMs with a mean DASH of 5 and a mean PRWHE of 7 show that the functional outcome of this technique is excellent. This is in line with the two other studies found measuring PROMs for intramedullary fixation of metacarpal fractures in general; Ozer et al. measured a mean DASH score of 9.5 (range 1–26) after 3 months and Schädel-Höpfner et al. for the fifth metacarpal neck fractures and measured a mean DASH score of only 0.8 (range 0–15) after 17 months [17, 18]. Nevertheless, using both PRWHE and the DASH questionnaire, as is done in our study, gives an even more precise idea of the functional outcome than shown in the existing literature.

This retrospective study must be assessed in the light of its limitations. Especially, the follow-up rate of 70% for the PROMs might introduce a type 2 error regarding functional outcome. Furthermore, the use of a tourniquet, the number of K-wires used, and the choice of aftercare were completely surgeon dependent. Although this lack of standardization does illustrate common clinical practice, optimal treatment could not be fully assessed. In line with common clinical practice as well and in favor of clinical feasibility, is the fact that the procedures were performed or supervised by six different surgeons. Due to their variable levels of experience but comparable outcome, this technique is generally applicable for most surgeons with interest in fractures of the hand.

## Conclusion

If surgical treatment for metacarpal shaft fractures is considered, we recommend antegrade intramedullary K-wire fixation with subsequent planned implant removal. This technique results in low complication rates and excellent functional outcome.

## References

[1] Court-Brown C, McQueen MM. Trauma (orthopaedic surgery essentials series). 1st ed. Philadelphia: LWW; 2006. pp 187–188

[2] Low CK, Wong HC, Low YP, Wong HP (1995). A cadaver study of the effects of dorsal angulation and shortening of the metacarpal shaft on the extension and flexion force ratios of the index and little fingers. J Hand Surg Br Scotl.

[3] Greeven APA, Bezstarosti S, Krijnen P, Schipper IB (2016). Open reduction and internal fixation versus percutaneous transverse Kirschner wire fixation for single, closed second to fifth metacarpal shaft fractures: a systematic review. Eur J Trauma Emerg Surg.

[4] Kozin SH, Thoder JJ, Lieberman G. Operative treatment of metacarpal and phalangeal shaft fractures. J Am Acad Orthop Surg (Internet). 2016;8:111–21. http://www.ncbi.nlm.nih.gov/pubmed/10799096. Accessed 1 Dec 2016 10.5435/00124635-200003000-00005 10799096

[5] Fusetti C, Meyer H, Borisch N, Stern R, Santa D, Della, Papaloïzos M. Complications of plate fixation in metacarpal fractures. J Trauma (Internet). 2002;52:535–9. http://www.ncbi.nlm.nih.gov/pubmed/11901331. Accessed 9 Nov 2016 10.1097/00005373-200203000-00019 11901331

[6] Page SM, Stern PJ. Complications and range of motion following plate fixation of metacarpal and phalangeal fractures. J Hand Surg Am (Internet). 1998;23:827–32. http://www.ncbi.nlm.nih.gov/pubmed/9763256. Accessed 1 Dec 2016 10.1016/S0363-5023(98)80157-3 9763256

[7] Foucher G, Chemorin C, Sibilly A. A new technic of osteosynthesis in fractures of the distal 3d of the 5th metacarpus. Nouv Presse Med (Internet). 1976;5:1139–40. http://www.ncbi.nlm.nih.gov/pubmed/934828. Accessed 1 Nov 2016 934828

[8] Corkum JP, Davison PG, Lalonde DH (2013). Systematic review of the best evidence in intramedullary fixation for metacarpal fractures. Hand (NY) US.

[9] Chammaa RH, Thomas PBM, Khalil A (2010). Single retrograde intramedullary wire fixation of metacarpal shaft fractures. Acta Orthop Belg Belgium.

[10] Zirgibel BJ, Macksoud WS. Self-correcting intramedullary Kirschner wire fixation of metacarpal shaft fractures. Tech Hand Up Extrem Surg. 2013;17(2):87–90. http://www.ncbi.nlm.nih.gov/pubmed/23689855. Accessed 1 Dec 2016 10.1097/BTH.0b013e3182821233 23689855

[11] Veehof MM, Sleegers EJA, van Veldhoven NHMJ, Schuurman AH, van Meeteren NLU. Psychometric qualities of the Dutch language version of the Disabilities of the Arm, Shoulder, and Hand questionnaire (DASH-DLV). J Hand Ther (Internet). 2002;15:347–54. http://www.ncbi.nlm.nih.gov/pubmed/12449349. Accessed 2 Nov 2016 10.1016/s0894-1130(02)80006-0 12449349

[12] Hudak PL, Amadio PC, Bombardier C, Beaton D, Cole D, Davis A, et al. Development of an upper extremity outcome measure: the DASH (disabilities of the arm, shoulder, and head). Am J Ind Med (Internet). 1996;29:602–8. Accessed 2 Nov 2016 10.1002/(SICI)1097-0274(199606)29:6<602::AID-AJIM4>3.0.CO;2-L 8773720

[13] El Moumni M, Van Eck ME, Wendt KW, Reininga IHF, Mokkink LB. Structural validity of the Dutch version of the patient-rated wrist evaluation (PRWE-NL) in patients with hand and wrist injuries. Phys Ther (Internet). 2016;96:908–16. http://ptjournal.apta.org/cgi/doi/10.2522/ptj.20140589. **(cited 2016 Nov 2)** 10.2522/ptj.20140589 26586862

[14] Orbay JL, Touhami A, de Jonge JJ, Kingma J, van der Lei B, Klasen HJ (1994). The treatment of unstable metacarpal and phalangeal shaft fractures with flexible nonlocking and locking intramedullary nails. Injury Engl.

[15] Muller MC, Welle K, Windemuth M, Burger C, Pennekamp PH (2013). Elastic titanium nails for minimally invasive intramedullary splinting of metacarpal fractures. Z Orthop Unfall Ger.

[16] Kelsch G, Ulrich C (2004). Intramedullary k-wire fixation of metacarpal fractures. Arch Orthop Trauma Surg.

[17] Ozer K, Gillani S, Williams A, Peterson SL, Morgan S. Comparison of intramedullary nailing versus plate-screw fixation of extra-articular metacarpal fractures. J Hand Surg Am (Internet). 2008;33:1724–31. http://www.ncbi.nlm.nih.gov/pubmed/19084170. Accessed 12 Dec 2016 10.1016/j.jhsa.2008.07.011 19084170

[18] Schädel-Höpfner M, Wild M, Windolf J, Linhart W. Antegrade intramedullary splinting or percutaneous retrograde crossed pinning for displaced neck fractures of the fifth metacarpal? Arch Orthop Trauma Surg (Internet). 2007;127:435–40. http://link.springer.com/. Accessed 12 Dec 2016 10.1007/s00402-006-0254-y 17123093

